# Ubiquitination of PHYTOSULFOKINE RECEPTOR1 regulates plant immunity

**DOI:** 10.1093/plphys/kiad224

**Published:** 2023-04-17

**Authors:** Shiqing Zhang, Jian Chen

**Affiliations:** International Genome Center, Jiangsu University, Zhenjiang 212013, China; International Genome Center, Jiangsu University, Zhenjiang 212013, China; Plant Physiology, American Society of Plant Biologists, USA

Plants use a two-tiered innate immune system to cope with the challenges of various pathogenic microbes (Jones and Dangl 2006). In the first layer of the immune system, cell-surface receptors recognize pathogen-associated molecular patterns or danger-associated molecular patterns (DAMPs), triggering pattern-triggered immunity (Chen et al. 2021b). The second layer of the immune system relies on nucleotide-binding leucine-rich repeat receptor proteins to detect pathogen effectors, resulting in effector-triggered immunity (Chen et al. 2021a). Plant-derived peptides can function as DAMP and activate intracellular immune signals. One of the well-studied DAMPs is phytosulfokine (PSK), a disulfated pentapeptide that promotes cell growth and alters immune responses depending on the type of pathogens (Sauter 2015). PSK is perceived by its receptor PHYTOSULFOKINE RECEPTOR (PSKR), a leucine-rich repeat receptor kinase (Wang et al. 2015). How PSKR1 is regulated at the protein level remains unclear. In this issue of *Plant Physiology*, Hu et al. (2023) found that tomato (*Solanum lycopersicum*) PSKR1 is regulated by the ubiquitin/proteasome degradation pathway (Hu et al. 2023).

**Figure 1.**
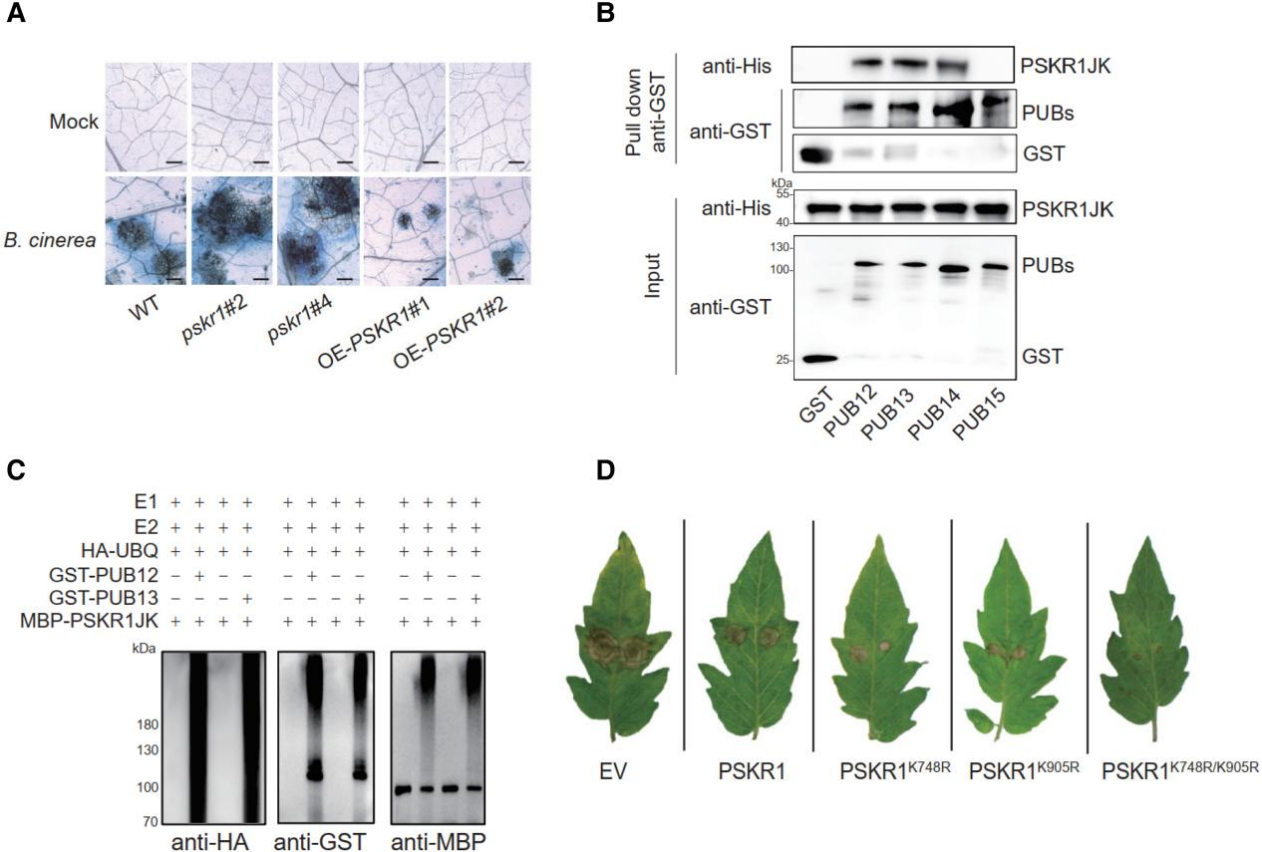
PUB12 and PUB13 interact with and ubiquitinate PSKR1. **A)** Trypan blue staining for cell death in *PSKR1* mutants and overexpressing lines inoculated with *B. cinerea*. **B)** PSKR1 interacts with PUB12/13/14 in a GST pull-down assay. **C)** PUB12/13 ubiquitinates PSKR1 in vitro. Ubiquitination of MBP-PSKR1JK was analyzed in the presence of E1, E2, ubiquitin, and GST-PUB12 or GST-PUB13. **D)** Effect of Lys-to-Arg mutated PSKR1 variants on plant resistance to *B. cinerea* in tomato OE-PUB13 lines.

In Arabidopsis (*Arabidopsis thaliana*), PSKR1 plays a negative role in plant defense against (hemi)-biotrophs, including the bacteria *Pseudomonas syringae* and *Ralstonia solanacearum* (Mosher et al. 2013). To investigate the functions of tomato PSKR1 in plant immunity, the authors generated *PSKR1* knock-out plants using CRISPR-Cas9 and overexpression (OE) lines by transgenic approaches. The authors found that *pskr1* mutants exhibited more severe disease symptoms than wild-type plants, whereas the *PSKR1*-OE lines displayed enhanced resistance to *Botrytis cinerea* (Fig. 1A) (Hu et al. 2023). Consistently, the expression of the defense genes *PHYTOALEXIN DEFICIENT3* (*PAD3*) and *SENESCENCE-ASSOCIATED GENE12* (*SAG12*) was inhibited in *pskr1* but increased in the OE lines, suggesting that tomato PSKR1 plays a positive role in defense response against *B. cinerea* in tomato.

It is well known that many receptor-like kinases can be degraded by the 26S proteasome pathway (Lu et al. 2011). The authors hypothesized that PSKR1 is also regulated at the posttranslational level. Indeed, the authors found that PSRK1 is subjected to the 26S proteasome degradation pathway but not the vacuolar degradation pathway. Previous studies showed that plant U-box (PUB) E3 ubiquitin ligases, PUB12 and PUB13, mediated the protein ubiquitination of multiple RLKs (Lu et al. 2011; Zhou et al. 2018). The authors postulated that tomato PUBs might also mediate the ubiquitination of PSKR1. Indeed, the authors found that PSKR1 interacts with PUB12, PUB13, and PUB14, but not PUB15 (Fig. 1B) (Hu et al. 2023). Furthermore, they found that PSK treatment weakens the interaction of PSKR1 with PUB12 and PUB13 but not PUB14. Together, these results suggest that PUB12/13 associates with PSKR1 to maintain the protein abundance and that PSK can reduce the interactions. The authors then determined the function of PUB12/13 and found that PUB12/13 functions as a negative regulator of plant defense against *B. cinerea* because *PUB12*- and *PUB13*-silenced lines showed enhanced disease resistance to *B. cinerea* compared with control plants (Hu et al. 2023).

Because PUB12 and PUB13 interact with PSKR1, the authors next investigated whether PSKR1 could be ubiquitinated by PUB12/13. Indeed, the authors found that PSKR1 was ubiquitinated in vitro and in vivo by PUB12/13 (Fig. 1C). Ubiquitination appears to affect the abundance of PSKR1 because PUB12/13 can degrade PSKR1 in planta. Furthermore, the authors found that PSK can inhibit the ubiquitination of PSKR1 by PUB12/13.

The authors further defined the exact ubiquitination sites: Lys748 and Lys905. They determined the roles of the 2 lysine residues through site-directed mutagenesis. Lys to Arg mutation disrupted the ubiquitination of PSKR1 and affected PSKR1 degradation. The authors then investigated the functions of Lys748 and Lys905 of PSKR1 in plant immunity. Lys-to-Arg mutated PSKR1 variants PSKR1^K748R^, PSKR1^K905R^, and PSKR1^K748R/K905R^ were transiently expressed in *Nicotiana benthaminana* leaves, which then were subjected to *B. cinerea* infection. *N. benthaminana* leaves expressing Lys-to-Arg mutated forms of PSKR1 displayed stronger resistance to *B. cinerea* than control plants (Hu et al. 2023). Similarly, tomato *PUB13-*OE lines transiently expressing Lys-to-Arg mutated forms of PSKR1 also showed increased disease resistance to *B. cinerea* (Fig. 1D).

In summary, Hu et al. confirmed that PSKR1 positively regulates plant immunity against *B. cinerea* in tomato. Importantly, the authors discovered that plant U-box E3 ligases PUB12 and PUB13 interact with PSKR1, and PUB13 causes PSKR1 ubiquitination at Lys-748 and Lys-905 to regulate the stability of PSKR1. However, PSK can attenuate the interaction between PSKR1 and PUB12/13 and reduce the ubiquitination. Overall, this study provides insight into the function of PSKR1 in plant immunity and posttranslational regulation of PSKR1 in the plant defense response. It is well known that RLKs are often regulated by phosphorylation (Mithoe and Menke 2018; Orosa et al. 2018). To gain a further understanding of the biological function of PSKR1, it will be worth testing whether PSKR1 is regulated by phosphorylation and sumoylation.
